# Successful Cases of Closed Reduction of Chronic Traumatic Atlantoaxial Rotatory Dislocation: A Report of Two Cases

**DOI:** 10.7759/cureus.36445

**Published:** 2023-03-21

**Authors:** Afif Abdul Latiff, Mohd Hisam Muhamad Ariffin, Navin Kumar

**Affiliations:** 1 Orthopaedics and Traumatology, Universiti Kebangsaan Malaysia Medical Centre, Kuala Lumpur, MYS; 2 Spine Surgery, Universiti Kebangsaan Malaysia Medical Centre, Kuala Lumpur, MYS

**Keywords:** c1-c2 fixation, atlantoaxial rotatory subluxation, atlantoaxial rotatory dislocation, closed manual reduction, traumatic atlantoaxial rotatory dislocation

## Abstract

We report two cases of children with atlantoaxial rotatory dislocation (AARD) post-trauma with a chronic history of persistent neck pain and torticollis. The neurological examinations were normal. The dislocation reduction was challenging; however, cases with such delayed presentation, treated with closed reduction and external stabilization, are rare. After reduction, a serial CT scan during follow-up showed no recurrence.

## Introduction

The causes of atlantoaxial rotatory dislocation (AARD) can be either traumatic or nontraumatic [1]. This condition can lead to complete paralysis due to proximal spinal cord compression if neglected and left untreated. There is still no consensus on the most effective way to treat AARD. Acute cases are usually treated with a cervical collar, whereas chronic refractory patients require halter or skull traction followed by immobilization via a halo vest or cervical collar [2]. We report two cases of successful closed manual reduction (CMR) of AARD followed by halo vest immobilization.

## Case presentation

Case 1

**Figure 1:**
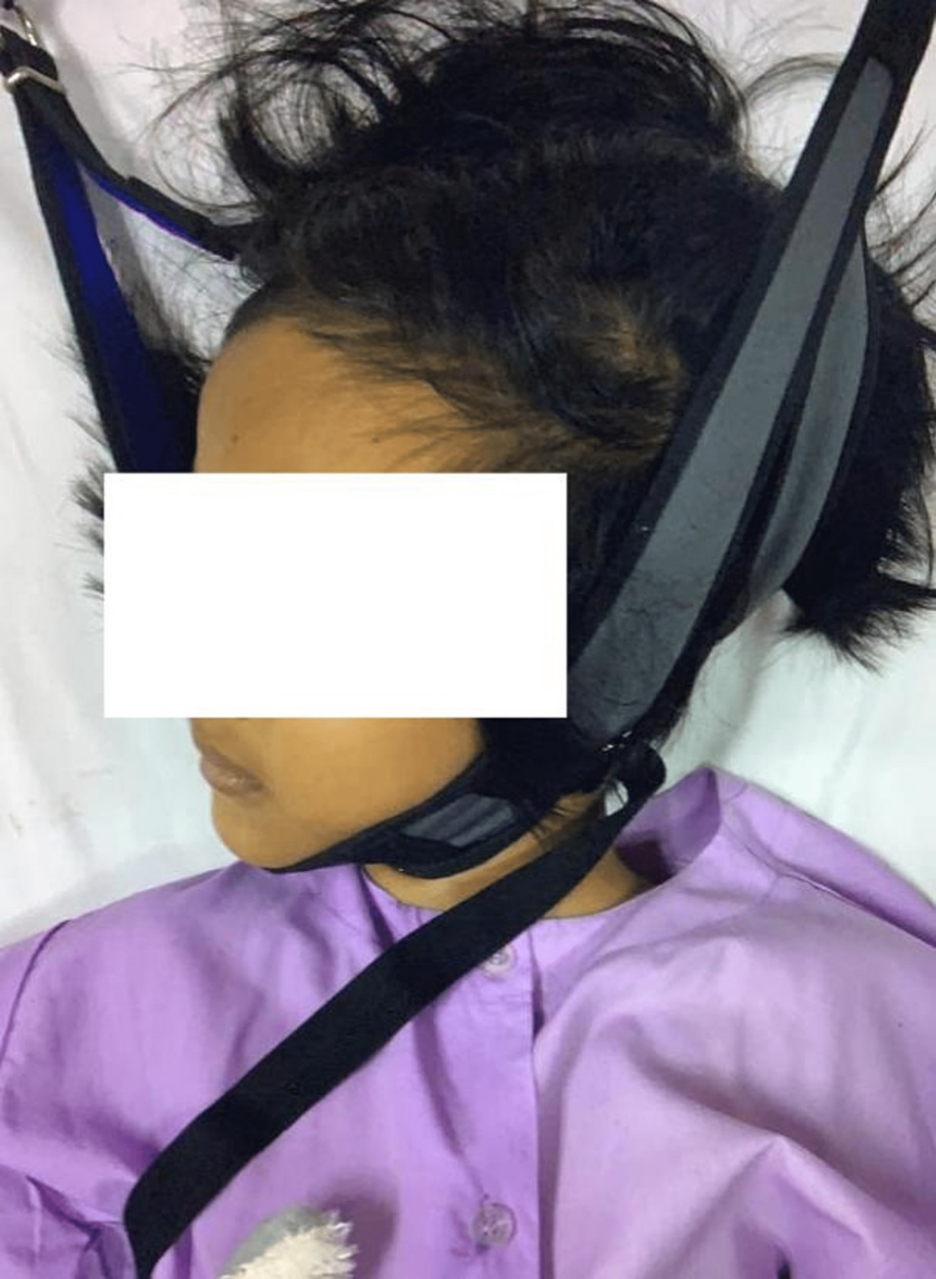
The child's clinical picture on halter traction with torticollis to the right side.

A six-year-old female with underlying bronchial asthma sustained atlantoaxial rotatory subluxation following a motor vehicle accident. She was initially treated at another hospital for her left shoulder dislocation and neck pain post-trauma. After three days of monitoring, she was discharged home. The child complained of persistent neck pain with torticollis (Figure 1) for five weeks post-trauma; thus, the parents decided to bring the child to the hospital.

The patient came to our center six weeks post-trauma complaining of persistent neck pain. She had no fever nor upper respiratory tract infection prior to the onset of the pain. On examination, her neck was tilted to the right side, and there were no syndromic features of the face noted. Her motor power, muscle tone, sensation, and reflexes were normal over bilateral upper and lower limbs. A CT scan of the cervical spine was done on the same day that revealed atlantoaxial rotatory subluxation (type II Fielding and Hawkins classification). Halter traction was fixed; it was started for one week with 3 kg and gradually increased to 5 kg. Close monitoring of her neurological function was done in the ward.

**Figure 2:**
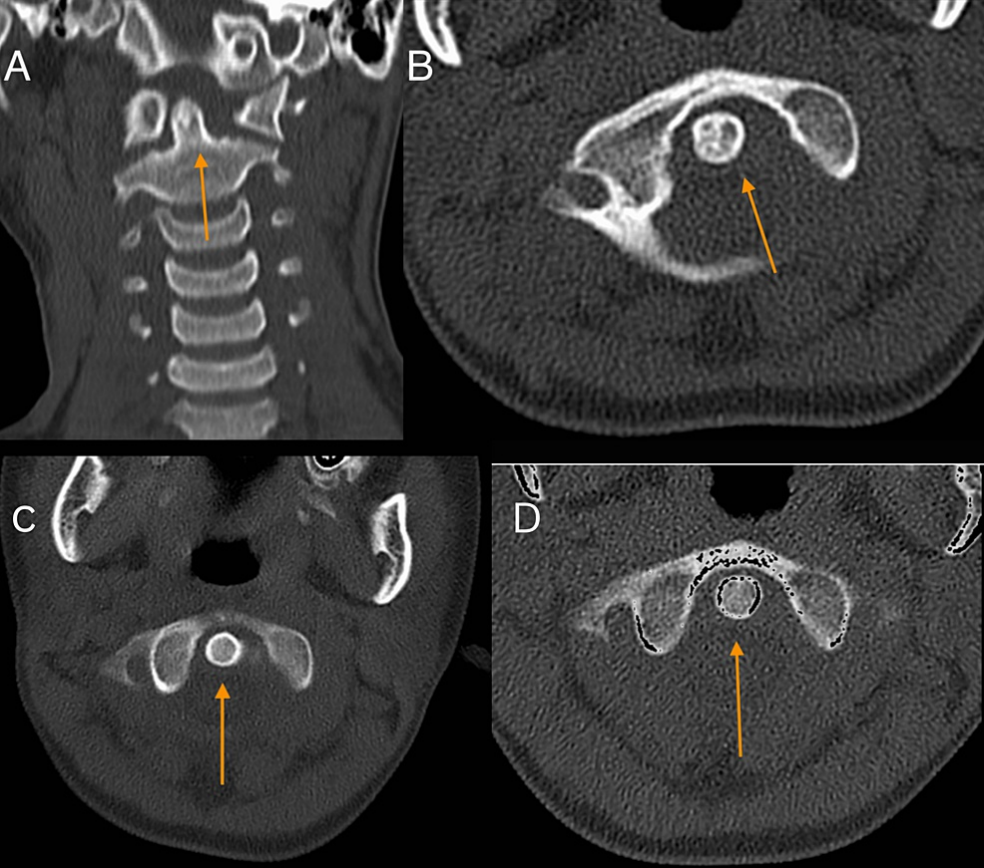
(A) Coronal and (B) axial cut section of the CT scan of the cervical spine eight weeks post-injury. It was noted that there was an improvement in the C1-C2 rotatory dislocation (arrow). (C) Axial cut section of the repeated CT scan two weeks post-closed reduction showed that the atlantoaxial rotatory dislocation improved (arrow). (D) The axial cut of the CT scan six weeks post-closed reduction showed a successful reduction (arrow). CT: computed tomography

After one week of halter traction, we noted persistent atlantoaxial rotatory subluxation evidenced by the CT scan shown in Figure 2. We decided to make a closed manual reduction (CMR) using Jeszenszky transoral technique [3]. Under general anesthesia, the surgeon fixed the spinous process of C2 using the nondominant hand while reducing the C1 by applying pressure transoral to the lateral mass of C1 anteriorly until the surgeon felt a "click." The electromyography was monitored throughout the procedure using neuromonitoring and showed no reduced readings. The alignment of C1-C2 was confirmed under image intensifier guidance. The patient was then put on halo traction for four weeks and then converted to a halo vest and was allowed to discharge home after reviewing the CT scan post-procedure as shown in Figure 2.

**Figure 3:**
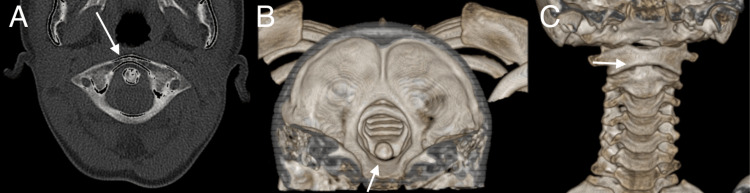
Repeated (A) axial cut of the CT scan and (B) axial and (C) coronal view of the 3D reconstruction CT scan of the cervical spine seven months post-removal of the halo vest showing no recurrence (arrows). CT: computed tomography

We followed up with the patient six weeks post-application of the halo vest in the clinic. We noted no more atlantoaxial rotatory dislocation from the repeated CT scan. The halo vest was removed four months post-closed reduction. We also repeated another CT scan seven months after we removed the halo vest, and there was no recurrence of atlantoaxial rotatory subluxation, as shown in Figure 3.

Case 2

**Figure 4:**
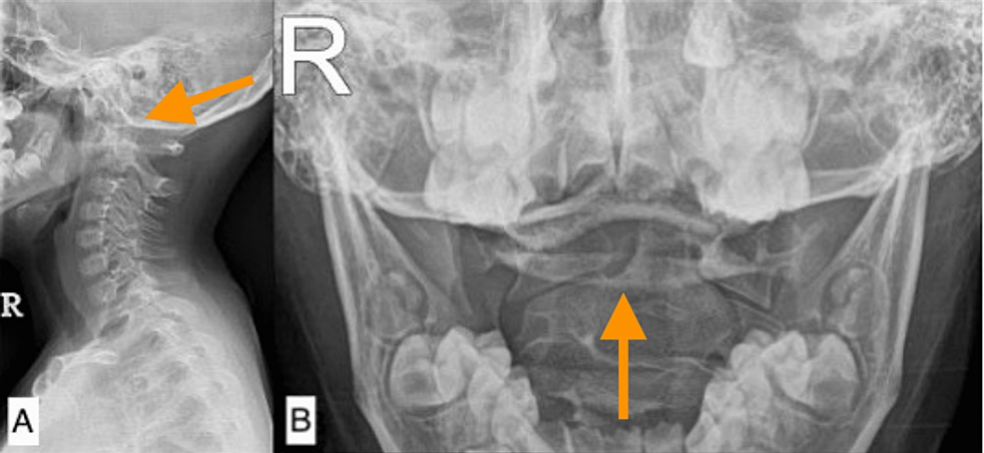
Pre-reduction cervical spine X-ray showing asymmetrical odontoid lateral interval and rotation: (A) lateral view and (B) open mouth view (arrows).

**Figure 5:**
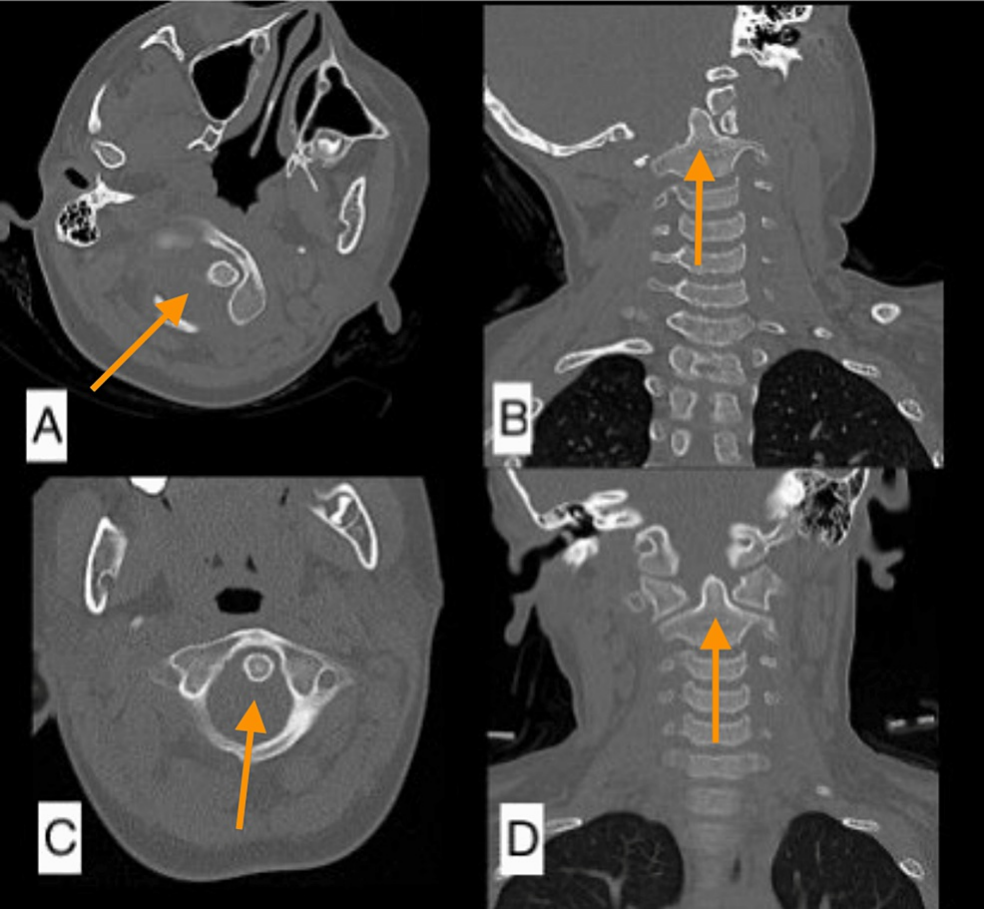
Pre-reduction CT cervical (A) axial view showing eccentrically positioned dens and (B) coronal view showing subluxation and rotation of the atlantoaxial joint complex (arrows) and post-reduction CT cervical (C) axial and (D) coronal view showing resolution of subluxation and restoration of alignment (arrows). CT: computed tomography

A nine-year-old female presented to our clinic with torticollis and neck pain of more than three months duration, which occurred after performing roly-poly at home. She had previously visited another hospital where she was diagnosed and treated for cervical muscle strain. She was neurologically intact, and other clinical findings were unremarkable. Her physical examination revealed torticollis in cock-robin position, in which the head was tilted to the right side of the neck and the chin rotated to the contralateral side. Cervical spine radiographs showed an asymmetrical interval between the atlas' lateral masses and the axis' odontoid, as shown in Figure 4. CT imaging revealed atlantoaxial rotatory subluxation, as shown in Figure 5.

**Figure 6:**
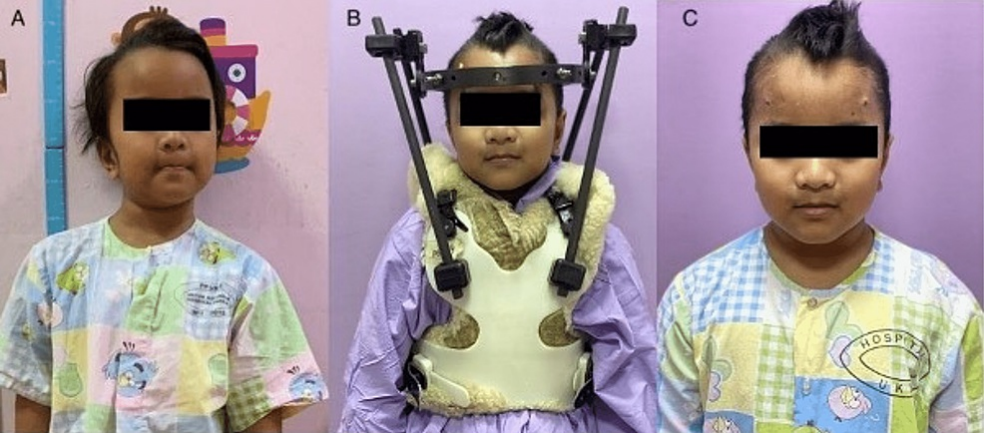
(A) Torticollis in cock-robin position on presentation. (B) Post-reduction in halo fixator. (C) Resolution of torticollis three months after the removal of the halo fixator.

The diagnosis of AARF (type II Fielding and Hawkins classification) was established with the given history, clinical findings, and imaging studies. The patient was treated with closed reduction and immobilization because the patient's neurology was intact, and no evidence of cervical spine instability was noted in imaging studies. The patient was subjected to a trial of closed reduction with halter traction, which was unsuccessful. Subsequently, the patient was managed with constant force halo crown traction fixed with four pins to the outer portion of the patient's skull, as shown in Figure 6, which was applied under general anesthesia in the operating theater. The traction continued with a gradual increase in weight (0.5 kg every two days) in a supine position for 10 days with a maximum weight of 5 kg.

Repeated cervical spine radiograph and CT post-successful reduction of deformity showed improvement in the degree of atlantoaxial subluxation, as shown in Figure 5. Finally, the halo ring was connected to the halo vest, and the patient was discharged home well. The reduction was maintained with a halo vest immobilizer for three months. After three months, repeat CT scan findings showed that the reduction was maintained, and the halo device was removed. The patient was started on gentle neck muscle strengthening exercises as tolerated. Follow-up six months later showed resolution of torticollis, as shown in Figure 6, and recovery of neck range of motion with no recurrence.

## Discussion

Traumatic rotatory subluxation commonly occurs in children than in adults. About 90% of cases involve those aged less than 21 years old. The factors that predispose it to happen in children are a giant head, underdeveloped neck musculature, a more lax joint capsule, the horizontal configuration of the atlantoaxial articular facets, and the interspinous ligaments that are elastic and flexible [1].

According to Fielding and Hawkins, the classification of an atlantoaxial rotatory subluxation is divided into four types. Type I is a simple rotatory displacement of the C1 and C2 cervical vertebrae without anterior shift with the transverse ligament intact, and dens act as a pivot point. Type II displacement involves rotation and an anterior displacement between 3 and 5 mm, the transverse ligament is injured, and the opposite facet acts as the pivot point. Type III is rotation and anterior displacement of more than 5 mm with both transverse ligament and facet capsules injured. A more severe type is type IV, a posterior subluxation of both lateral atlantoaxial joints. The most severe type is a frank rotatory atlantoaxial dislocation [2,4].

The treatment guideline traditionally proposed according to Fielding and Hawkins's classification is as follows [4]: type I may be treated with cervical spine immobilization after reduction; type II may be treated with halo traction, and if the reduction is achieved within 14 days of luxation in children, subsequent immobilization with a halo vest; and type III and IV lesions are treated with open reduction and posterior arthrodesis.

The time of presentation post-injury that took longer than 24 hours comprises about 20% of children with AARS. This may happen because there was only minor trauma or it was mistakenly diagnosed as a muscle spasm [5]. Therefore, a higher degree of suspicion in diagnosing AARD is required as Phillips and Hensinger suggest that the treatment plan should be based on the duration of the lesion. Patients who have had the symptoms for less than a week can be treated with soft collar immobilization and bed rest for one week. If there is no spontaneous reduction, the patient should be hospitalized and put on traction. They also recommend that in patients who have had the lesion for more than one week but less than a month, there is no role for a soft collar, and the patient needs to be on cervical traction. After reduction, four to six weeks of immobilization and follow-up are recommended. However, an attempt at cervical traction can be considered in patients with the symptoms for more than a month, but if reduction does not occur within three weeks, an arthrodesis should be performed [4].

A case of a closed reduction on type II atlantoaxial rotatory subluxation was treated with a closed reduction in which, in that particular case, rotatory unlocking and the counter-rotating maneuver were done with a transoral displacement of the subluxated C1 lateral mass using finger pressure along the posterior pharyngeal wall. However, the surgeon proceeded with instrumentation due to suspicion of noncompliance [6].

For a patient with symptomatic atlantoaxial dislocation, surgical intervention is agreed widely to prevent progressive neurological symptoms, respiratory failure, and death. However, the decision on surgical intervention for asymptomatic atlantoaxial dislocation is still debatable. Patients diagnosed after three weeks of the symptoms with failure of conservative treatment have a greater risk of recurrence, possibly due to chronic changes to the transverse and alar ligament, and are suggested for surgical treatment. However, the recurrence rate is still not well established. The use of internal fixation in children under six years is less recommended due to smaller ligament size [7].

In cases with no spontaneous reduction within three weeks, the closed reduction under mild sedation is also suggested with manual traction in minimal flexion, followed by cervical extension and contralateral rotation [7]. Another recommended technique is locking the spinous process of C2 with one hand posteriorly and pressing on the lateral mass of C1 using the other hand's index finger anteriorly through the posterior wall of the oropharynx through the Jeszenszky transoral technique as what we have done on our patients [3].

## Conclusions

Atlantoaxial dislocation is a fatal condition, if not corrected. Delaying treatment of the lesion may result in a higher risk of respiratory failure, neurological symptoms, and death. Surgical treatment is indicated in symptomatic atlantoaxial dislocation and failure of conservative treatment. However, the closed reduction has a role in chronic C1-C2 dislocations of more than three weeks.

## References

[1] Barimani B, Fairag R, Abduljabbar F, Aoude A, Santaguida C, Ouellet J, Weber M (2019). A missed traumatic atlanto-axial rotatory subluxation in an adult patient: case report. Open Access Emerg Med.

[2] Spinnato P, Zarantonello P, Guerri S (2021). Atlantoaxial rotatory subluxation/fixation and Grisel's syndrome in children: clinical and radiological prognostic factors. Eur J Pediatr.

[3] Jeszenszky D, Fekete T, Kleinstück F, Haschtmann D, Loibl M (2018). Transoral closed reduction of fixed atlanto-axial rotatory-subluxation (AARS) in childhood and adolescence. Clin Spine Surg.

[4] Goel A (2019). Torticollis and rotatory atlantoaxial dislocation: a clinical review. J Craniovertebr Junction Spine.

[5] Powell EC, Leonard JR, Olsen CS, Jaffe DM, Anders J, Leonard JC (2017). Atlantoaxial rotatory subluxation in children. Pediatr Emerg Care.

[6] Opoku-Darko M, Isaacs A, du Plessis S (2018). Closed reduction of traumatic atlantoaxial rotatory subluxation with type II odontoid fracture. Interdiscip Neurosurg.

[7] Barcelos AC, Patriota GC, Netto AU (2014). Nontraumatic atlantoaxial rotatory subluxation: grisel syndrome. Case report and literature review. Global Spine J.

